# Why ethnicity and gender matters for fertility intention among married young people: a baseline evaluation from a gender transformative intervention in rural India

**DOI:** 10.1186/s12978-018-0500-0

**Published:** 2018-04-13

**Authors:** Tina Khanna, Murari Chandra, Ajay Singh, Sunil Mehra

**Affiliations:** MAMTA Health Institute for Mother and Child, B-5, Greater Kailash Enclave-II, New Delhi, 110048 India

**Keywords:** Tribal, Fertility, Young men and women, Gender, Education, India

## Abstract

**Background:**

Social inequities in early child bearing persist among young married people, especially among tribal populations in India. Rural women belonging to tribal groups and those coming from poor households are more likely to give birth before age 18. This paper explores the connection between ethnicity, gender and early fertility intention among young married people in rural India.

**Methods:**

The data is drawn from a cross sectional baseline evaluation of an intervention programme in rural India. A sample of 273 married young people was taken. Respondents were selected using systematic random sampling. Logistic Regression was used to assess the effect of being a tribal on early fertility intention and also to determine if covariates associated with early fertility intention differed by tribal status. Qualitative data was analysed using deductive content analysis approach.

**Results:**

Bivariate and logistic regression results indicated that young married people from tribal communities had higher odds of planning a child within one year of marriage than non-tribals (OR = 1.47, *p*-value-0.079). Findings further suggest that early fertility intention among tribals is driven by gender factors and higher education and among non-tribals, higher education and awareness on contraception are key predictors. Among tribals, the odds of planning a child within one year of marriage was strongly associated with inequitable gender norms (OR = 1.94, *p*-value-0.002). Higher education showed significant positive association with non-tribals (OR = 0.19, *p*-value-0.014) and positive association with tribals (OR = 0.56, *p*-value-0.416). Qualitative investigation confirms that fertility desires of young married people are strongly influenced by gender norms especially among tribal populations.

**Conclusion:**

Early child bearing was underpinned by complex ethnic factors and gender norms. Preference for early child bearing was seen most among tribal communities. Gender attitudes were a cause of concern especially among tribal groups. These results suggest that efforts to improve early child birth will require changing gender norms related to fertility among tribals as well as social equity issues including higher education among non-tribals and tribals.

## Plain English Summary

Despite increase in age of marriage and declining fertility, social inequities in early child bearing exist among young married women and men, especially among tribal populations in India. This study sought to understand ethnic variation in early fertility preference among young people in rural India. This study utilizes mixed method approach to assess the effect of being tribal on early fertility intention and also whether determinants associated with early fertility intention differed among tribals and non-tribals. The data was collected through a survey of young married women (ages 14–18) and men (ages 15–21) and also through qualitative interviews with young married women and men, community health workers and parents. Results showed that preference for early child bearing is significantly associated with tribal communities who had early marriages and low contraceptive use. This is attributable to inequitable gender norms associated with early child bearing among tribals. Also, the role of higher education in influencing fertility intention among non-tribal and tribal cannot be neglected. The analysis underscores the need to address gender inequitable attitude among tribals and to amplify efforts to promote higher education among tribals and non-tribals for delaying early child birth.

## Background

In India, rates of early childbearing are persistently high, especially among ethnic and caste minorities and women from socio-economic disadvantaged backgrounds. Analysis of the National Family and Health Survey (NFHS-3) data indicates that rural women - those belonging to Scheduled Tribes (ST), and those coming from poor households were more likely to have had a birth before age 18 [1]. Fertility rates are higher for rural women in STs in comparison to Scheduled Caste (SCs), Other Backward Classes (OBCs), and general category [2]. Couples in tribal communities report some of the lowest rates of contraceptive use in India [2, 3].

Despite India’s recent economic growth, health and human development indicators of STs or *Adivasi* lag behind the national averages [4, 5]. Among the social groups in India, tribes are the most socio-economically deprived groups with low literacy and poor economic conditions and low access to health services [4–6]. The latest NFHS-4 (2015–2016) data also highlighted that reproductive health services are least accessed by women from ST groups. Tribal women also had lower rates of antenatal care visits and institutional births among all social groups [7].

Although there is extensive literature on various aspects of population, fertility, and family planning, it is noteworthy that only a limited number of empirical studies have attempted to explain tribal reproductive health (especially among young people) systematically in India [8–10]. A recent study of adolescent fertility in Africa highlights that first birth among young people is most common among the poorest and least educated, and progress in reducing rates within this group has not been made over the last few decades [11]. It further highlighted that it is valuable to examine fertility behaviour of different cultural groups to target programmes at the people most at risk, in order to reduce young people’s fertility [11].

A few micro-level studies in India demonstrate that being from a tribal community positively influenced the probability of early child bearing. Panel data from Andhra Pradesh [12] demonstrates that young married women from the tribal groups were 10.5% more likely to give birth by the time they were 19 years old than women from other castes [12]. Some studies in India highlighted that early age at marriage, son preference, lower education, and low access to services are some of the reasons for low contraceptive use among tribals [13]. Gender norms are an important aspect of early fertility desires, which are largely overlooked in interventions, especially for marginalised sections. Gender relations among Indian tribes have historically been more balanced and equitable; however there is an increasing trend of gender bias in tribal culture emerging due to the assimilation and modernising process [14]. In consonance with the recent declines of tribal female-male ratio (FMR) there is growing evidence to showcase the disadvantages that tribal females have been undergoing due to expansion of developmental activities and integration. A recent study suggests that the sharp drop in tribal FMR is due to assimilation into the patriarchal norms of higher castes through ongoing processes of sanskritisation and detribalisation -all contributing to an emerging culture of discrimination against women among tribal people [15, 16]. Therefore, the present paper tries to understand reproductive behaviour among young married people of tribal communities in India and explore the inter-connection between ethnicity and gender with early fertility intention, an important factor that has not been well established or investigated systematically.

The specific objectives of the paper are: (a) To explore fertility intention among tribal and non-tribal young married people (b) To assess the effect of being a member of tribal community on early fertility intention among young married people. (c) To assess gender and social determinants of early fertility intention among tribal and non-tribal young married people in rural India.

## Method

### Study design

This study involved analysis of cross-sectional baseline (using quantitative and qualitative methods) data from young married people^1^ in a family planning evaluation study in rural India. This paper is based on the analyses of young married people living in marital home aged 14 to 21 (Total = 273, tribal = 108, non-tribal = 165). The survey was conducted in two states of India – Madhya Pradesh (MP) and Rajasthan (two districts each) where early marriage (55–60%) and total fertility rate in rural areas fertility was higher (3.3- MP and 3.6- Rajasthan) than national estimates (National Family Health Survey, 2007–08). A three-stage sampling design was implemented in selecting six blocks across study districts and further from each blocks, 10 villages were selected using Probability Proportion to Size. Systematic random sampling was used to randomly select the respondents from completed household list of selected villages.

### Data

The quantitative data was collected through a structured questionnaire by trained male and female field investigators, taking an average time of 40 min. Male investigators interviewed young married men, and young married women were interviewed by female investigators. Survey data assessed determinants of fertility intention among these people by tribal and non-tribal groups. The survey tool contained questions on socio-demographic information (including age, sex, ethnic group, education, age at marriage) and also on fertility intention, decision making, gender attitudes, use of contraceptives etc. The instrument was verbally translated into Hindi and was field-tested to ensure language and cultural contexts.

### Qualitative methods

A total of 40 in-depth interviews (IDIs) were conducted among young married men and women (10 each) and parents (10 each - father and mother). We also conducted key informant interviews (KIIs) (8) with health care providers. Under the National Rural Health Mission, the government of India has employed Accredited Social Health Activist (ASHA) and Anganwadi workers (AWW) who act as community health workers. They provide counselling to married couples for use of family planning methods and also support community to access public health services. ASHA and AWW were purposively selected for KIIs since they are first point of contact with young married people in village for health care counselling and services including family planning.

We included perspectives of both young men and women and parents of young men and women to better understand emergence of early fertility norms within their particular social environments. For IDIs it took approximately 60 min and around 40 min for KIIs. The qualitative instruments contained questions relating to cultural norms and practices around early marriage and fertility, knowledge, access and use of family planning methods, gender barriers, etc. Purposive sampling was followed to select participants for IDIs and KIIs. We selected married young girls (aged 14–18) and married young boys (15–21) who were married in last 1 year without any children/not currently pregnant (married young women and wife of married young men) from tribal and non-tribal communities. For the health care providers, we recruited community health workers who had more than two years of working experience in the community. The senior research team (qualitative) in collaboration with field team with extensive experience conducted interviews.

### Ethics, consent and permissions

All study participants provided consent and/or assent to participate in the study. Verbal informed consent of parents, health care providers, young men and women was sought prior to the survey. Respondents aged below 18 provided assent, while verbal informed consent was sought from their parents. The questionnaires did not request the name or other identifying variables, to ensure anonymity of data for research purpose only. Participants did not receive any material compensation. Ethical permission was obtained from the Institutional Review Board (IRB) of MAMTA- Health Institute for Mother and Child (India) prior to data collection. Since young men and women aged 14–17 years are the most vulnerable populations in research, hence the study minimised all possible risks to the participants during the survey. Every effort was made to ensure protection and confidentiality and to reduce any potential adverse consequence to the participants.

### Measurement

Outcome variable: The response (dependent) variable was chosen to investigate early child bearing plan among married young people. This was categorised into two levels: intention to have child within one year of marriage and after one year. Hence, the dependent variable was coded dichotomously within one year (coded as 1) and after one year (coded as 0).

### Explanatory variables

Selected socio-demographic factors that may affect early child plan at young ages were controlled in an adjusted model. These variables were: gender of respondent, age at marriage, age at *gauna*, caste, education and economic status and gender attitudes. Since the data consists of young married men and women, hence fertility choices by genders was taken in an adjusted model. *Gauna* is a traditional custom in rural India (performed in marriage that occurs at very young age) where a married couple consummate their marriage and cohabit together. Education level was grouped as till primary level (0–5 years), secondary (6–10 years); and senior secondary and higher (11 years and above). Economic status was measured by household having Below Poverty Line (BPL) card or Antyodhaya card (extreme poor) provided by Government of India. Social-ethnic group was grouped/categorized as tribal (STs) and non-tribals (OBCs and SCs). Awareness of modern contraceptive method such as condoms, IUD, pills and injectable was computed. Anganwadi centers (AWC) is the nearest and the most feasible place that provides basic health care in Indian villages including contraceptive counseling and supply and reproductive health education hence awareness about AWC was coded as 1 and 0 otherwise. Exposure to mass media (measured as mobile/internet and books/magazine) was also computed.

Gender attitude of young married people was measured through Gender Equitable Men Scale (GEMS) on 10 statements related to early child bearing, contraceptive use, family planning, autonomy, reproductive rights, etc. The statements were a mix of attitudes that were gender equal and non-equal. For each statement, the response options were ‘agree’, ‘partially agree’ and ‘do not agree’. All the statements were made unidirectional before assigning the scores to a gender discriminatory statement. A score of 0 was given to the most negative statement and a score of 2 was assigned to the most positive statement. Factor analysis was done using Principle Component Analysis (PCA) and factor loading was performed to identify the most important variables with highest variability explained (Eigenvalues) in particular factor (index) created with higher reliability value (α = 0.81). Hence the predicted scores on the scale were distributed in three equal fragments assigning as low, moderate and high gender equitable attitudes. Of these, the two very relevant statements were chosen after bivariate tests were used in an adjusted model. This methodology of creating a GEM scale has been used and validated in previous research in India as well as other countries [17, 18].

### Statistical analysis

Bivariate analysis and Chi square assessed associations between tribal status and selected socio-demographic variables, early fertility intention, contraceptive use and gender norms. Adjusted Logistic Regression was used to assess effect of tribal status on early fertility intention. Intention to conceive after one year was taken as a reference category. Further, separate models were developed to determine if covariates associated with early fertility intention differed by tribal status.

### Qualitative analysis

For the qualitative data analysis, all the interviews were recorded, transcribed and subsequently translated into English. Professional translators well versed with English and Hindi translated the Hindi transcript into English. The first author (Indian speaking English and Hindi) cross checked all English translated transcripts with hindi transcripts (also checked with the audio files whenever necessary) to ensure quality of translation. Hindi transcripts were read several times to get acquainted with raw data and checked at random by the first author. The translated text was coded inductively, and ambiguities in meaning were resolved by consulting project staff. Data were organised using Atlas-ti software following a content analysis approach. Themes emerging during review of the transcripts were sorted and grouped according to key categories.

## Results

### Socio-economic characteristics, education and exposure

Participants (*n* = 273) were 39.5% tribal and 60.5% non-tribal. The mean age for tribal and non-tribal young people were 18.7 and 18.6 The socio-demographic characteristics varied among tribal and non-tribal groups. The majority of young married tribal people (77.8%) belonged to either poor or extremely poor families. Compared to non-tribal groups, young people from the tribal community received less education. More than 40% of the young tribal people had either no education or only primary education compared to nearly one-fourth of young non-tribal people. Only about 17% of the young tribal people reached education levels above class 10th as against one third of young non-tribal people (33.3%). The exposure to media through mobile/internet and books/magazine was also very low among tribal as compared to non-tribal people. Young tribal people (55.6%) had less access to mobile and internet than their non-tribal counterparts (61.8%).

### Early marriage, fertility preference and contraceptive use: Bivariate analysis

Tribal status of young people was significantly associated (*p* < 0.001) with younger age at marriage; and younger age at cohabitation with spouse *(Gauna)*. Around half of tribal young women (46.6%) were married before age 16 in comparison to non-tribal (27.1%). Also, the majority of young tribal men (63.5%) were married before age 18 as compared to nontribal (46.4%). Importantly, 23.3% of young tribal women and majority of young tribal men (42.8%) start cohabiting as married couples before age 16 and 18 years respectively in comparison to non-tribal young women (12.9%) and non-tribal young men (24.6%) as reflected in Table 1.

**Table 1:** Socio demographic and family planning characteristics among tribals and non-tribals

	Tribal (*n* = 108)	Non-tribal (*n* = 165)	*p*-value
Mean age*	18.7 *.54	18.6 *.53	
Socio-Economic characteristics			
Below Poverty Line and extreme poor	77.8	52.1	0.001
Education			0.002
None	15.7	9.1	
Primary (1-5th standard)	25.9	13.3	
Up to Secondary (6-10th Standard)	41.7	44.2	
Higher secondary & above (10th Standard and above)	16.7	33.3	
Exposure to media			
Mobile/ Internet	55.6	61.8	0.303
Books/magazines	5.6	21.8	0.001
Marriage			
Young women married below 16 years	46.6	27.1	0.001
Young men married below 18 years	63.5	46.4	0.001
Age at *gauna* (young women) below 16 years	23.3	12.9	0.001
Age at *gauna* (young men) below 18 years	42.8	24.6	0.001
Family planning characteristics			
Plan to have child within one year of marriage	27.4	18.7	0.088
Awareness of modern contraception methods: condom	46.3	55.4	0.152
Awareness of modern contraception Methods: Pills	82.4	93.9	0.002
Awareness about place to get contraceptives near village (AWC)	27.8	45.4	0.003
Currently using any method	15.7	24.2	0.091
Methods of use (modern method)	14.8	23.0	0.096
Reasons for not using contraception			0.053
Lack of knowledge of modern contraceptive methods	24.7	14.1	
Planning to conceive	55.3	53.9	

*Standard deviation

Data on fertility preference indicate a significant association between tribal status and intentions for early child bearing. Within the study sample, 27.4% of young married tribal people expressed the desire for having a child within one year of marriage compared to 18.7% of their non-tribal counterparts. A difference was also observed on awareness of modern contraceptive methods (condoms and pills) between tribal and non-tribals. Also, less percentage of young married people who belonged to tribal families (27.8%) were aware of AWC as the place within or near the village where one could get modern contraceptives in comparison to non-tribals (45.4%). Likewise, the current contraceptive (modern method) use among tribals (15.7%) was less than among non-tribals (24.2%).

### Gender Attitudes towards early child bearing among tribal and non-tribal young married people

Tribal and non-tribal young married people were asked whether they agree, partially agree or disagree with specific gender statements related to early child bearing which provides deeper insight into the effect of gender-related attitudinal differences among tribal and non-tribal young people, on fertility intention. Gender attitudes of young married people and especially in tribal groups were biased against women when it comes to planning for the first child. As reflected in Table 2, a vast majority of young married tribal people (68.5%) agreed to the statement that ‘*it is husbands who can decide when to have a first child’* in comparison to 50.3% of non-tribal young people. There was a difference in the attitudes of young people, especially among tribal groups towards the pressure of early child bearing for young women, with tribals displaying more rigid and biased attitudes. Half of young tribal people agreed that ‘*women should conceive within one year of marriage to avoid shame*’ in comparison to only 31.5% non tribals. Also more than half of young tribal people were in agreement with the statement ‘*Early child bearing makes women valuable in family and society’,* than their non-tribal counterparts (37%).

**Table 2:** Gender attitudes on early child bearing (percent who agreed to selected statements) among tribals and non-tribals young married people

Statements	Tribal %	Non-tribal %	*P*-value (Chi-square)
If a couple doesn’t have a child then it’s a woman’s fault	27.8	18.2	0.061
It’s a woman’s responsibility to avoid getting pregnant	59.3	51.5	0.209
Women should conceive within one year of marriage to avoid shame	50.0	31.5	0.002
It is husbands who can decide when to have first child	68.5	50.3	0.003
The real man is the one who has child within one year of marriage	23.2	16.9	0.207
Early child bearing makes women valuable in family and society	51.9	37.0	0.015

**Fig. 1:**
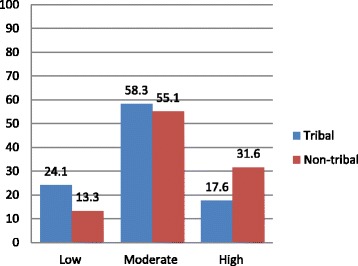
Level of support for gender equity among tribal and non-tribals: Fertility Perception on Early Child Bearing (separate file attached)

Further, a Gender Equitable Men Scale (from the individual statements) was developed (as explained in methodology section) whereby individual scores of members were placed in one of three categories (low, moderate and high) reflecting their level of support for gender equity. The GEMS scale (Fig. 1) shows that a majority of young tribal people fall into low and moderate gender equity category, meaning that they hold attitudes that were less or moderately supportive of gender equity towards early child bearing (*p* < 0.001). As seen in Fig. 1, young non-tribal people were more gender equitable than their tribal counterparts.

### Multivariate Analysis: Logistic regression (adjusted Model) for combined and separate ethnic groups

The logistic regression analyses showed that tribal status was a significant predictor for intention to have early child birth while adjusting with other confounders (Table 3). Young people from tribal communities had 47% higher odds to plan a child within one year of marriage than their non-tribal counterparts (OR-1.47, *p* < 0.079). In the combined model, gender measures (norms) and education were other significant predictors. Young married people with restrictive gender attitudes had higher odds to plan a child within one year of marriage. For example, young married people who agreed with the statement *‘The real man is the one who has a child within one year of marriage’* had 78% higher odds of planning a child within one year of marriage than those who disagreed with the statement (OR = 1.78, *p* < 0.004), given that the other predictors in the model held constant. Also, overall, the young married women had higher odds to plan a child within one year of marriage as compared to men (OR-1.55, *p* < 0.067). Young married people with higher level of education (senior secondary and higher), had lower odds to go for early child bearing (both tribals and non-tribals in the combined model) than those who had low level of education (OR-0.39, *p* < 0.025).

**Table 3:** Odds Ratio (OR) of Fertility Intention within one year by selected predictors for combined and separate ethnic groups

	Model 1: Combined ethnic groups	Model 2: Tribal	Model 3: Non-tribal
	OR	OR	OR
Gender			
Boy	1	1	
Girl	1.55*	1.82**	1.63
Caste			
Non-tribal	1	1	
Tribal	1.47*		
Education level			
Up to primary (0-5th standard)	1	1	1
Secondary (6-10th Standard)	0.88	0.80	0.93
Senior secondary & higher (10th standard and above)	0.39**	0.56	0.19**
Duration of cohabitation after marriage (years)	1.00	1.20	0.95
Awareness about contraception methods:			
Yes	1	1	1
No	1.52**	1.46	1.71**
Gender statements			
The real man is the one who has a child within one year of marriage			
Disagree	1	1	1
Agree	1.78***	1.94***	1.33
Early child bearing makes women valuable in family and society			
Disagree	1	1	1
Agree	1.08	0.37	1.42
Constant	0.015	0.0666	0.031
Adjusted R^2^	0.115	0.2101	0.181

Note: Response outcome for early fertility preference 0(ref) = after 1 year, 1 = within 1 year, results are controlled for other predictors such as current age, BPL status, family support @reference category. *p < 0.1, ** *p* < 0.05, ***p < 0.01

Further, separate models were developed to determine if covariates associated with early fertility intention differed by tribal status. Among tribal groups, early fertility intention was significantly associated with gender factors unlike their non-tribal counterparts. Young people from tribal group who agreed with the statement ‘*The real man is the one who has a child within one year of marriage*’ had 94% higher odds of early child bearing than those who disagreed with the statement (OR-1.94, *p* < 0.002). Within the tribal group, women had 82% higher odds of planning a child within one year of marriage as compared to their male counterparts (OR-1.82, *p* < 0.020).

Early fertility intention was positively associated with increase in education among both tribal and non-tribal groups. Table 3 reflects that with increase in level of education the odds of planning a child early after marriage decreased irrespective of ethnic groups. Among non-tribals higher education had positive and significant association (OR-0.19, *p* < 0.014) with early fertility intention, however among tribals it showed only positive association (OR-0.56, *p* < 0.416). Among non-tribals, young married people who were not aware of contraceptive methods had 71% higher odds of planning a child within one year of marriage (OR-1.71, *p* < 0.026). Thus among non-tribals, early fertility intention was significantly associated with education and awareness about contraception and not with gender factors.

### Qualitative findings

The qualitative investigation found that the desire for young people to bear children early was influenced by social and gender norms, especially among tribal populations.

### Social and family related factors

The pressure to fulfill the social demand to conceive early to prove their worth soon after marriage was found among young women, and this was particularly high for tribal women who were married early and had less education. Parents from both tribal and non-tribal communities shared that it is a common trend for a married couple to have a first child early, and therefore no birth control measures were taken. They were taken only after the second child for spacing. However, in the context of tribals, parents and families were more averse towards imparting family planning to newly married couples and feared using family planning methods. These respondents (parents) also had less education (primary education) and less exposure. They shared that talking about sexual relations, family planning issues is neither needed nor acceptable in the community. It is considered a cultural taboo and some parents even feared and questioned the relevance of providing this knowledge. As shared by an elder woman from the tribal community:

“*No one talks about these things in a village. It is considered a wrong and sinful practice. You should not give this information otherwise newly married will know about ways to delay pregnancy and they will delay their first child. You should give this information to those who have at least one child.”*

Young married tribal women shared that there is strong family pressure on them to conceive early especially for the first child. They explained that they have limited decision-making powers in family planning; these decisions are mostly taken by the husband. As shared emphatically by a married tribal girl (19):

*I: What will you do if you don’t want to have a child now?*

*R: I/my husband will have to use birth control methods.*

*I: Will you face any difficulty in using contraceptives?*

*R: My in-laws can stop me; they will say we want to have a grandchild now.*

*I: What will you do? What support would you need?*

*R: I would definitely need my husband’s support to refuse to have a child now. If my husband wants it, then I will have to conceive and deliver a child, and if he doesn’t then I will not conceive.*

Young men were also influenced by social norms about early fertility and perceived attitudes of people in their social networks. Young married men also stated that there are negative repercussions that a woman has to bear if she doesn’t conceive within two years of marriage, including facing negative remarks and abandonment. This was shared by both tribal and non-tribal men. The young tribal men who were married early (at 16–17 years) and had less education, were found more bound by traditional community norms for early child bearing.

*“It is expected to have a child within one year of marriage. If she doesn’t conceive within 1-2 years then such a woman is taunted, taken to doctors and may be abandoned.”*

Married boy (19 years), Scheduled Tribe.

Among tribal populations, around two-thirds of interviewed young married tribal men and women (*n* = 6) lacked correct knowledge about pregnancy. Few of them thought that pregnancy can never occur in one sexual contact and it is possible after multiple contacts. The knowledge about use of contraception was also less among married tribal young people as less than half of them were aware of it (*n* = 4). Most of these respondents were married at the age of 15–16 years and had education less than higher secondary. As shared by a young married tribal man (18 years): “*No I have not used a condom, and I also don’t know how to use it. In fact, I will never use; it looks horrible to me.”*

### Factors related to service provision/health care providers

The qualitative interviews with health care workers highlighted their apprehension to provide family planning knowledge and services to young married men and women and their limited outreach to tribal communities. Many village health care providers expressed disapproval at using contraceptive methods to delay the first pregnancy and spoke of community challenges and the non-acceptability of it.

“*The first child should be born after two years - we can counsel the couple on this but we can’t go against society. Even if the couple is educated and aware, they will be forced to have the first child soon. They can delay the second child not the first.”*

Community health worker (Female, 34 years).

Community health workers also expressed that young married men and women from tribal communities lacked awareness and access to health care services. They explained that they were not able to reach them as they lived on the periphery of the village, far from the health centre.

T*ribal communities live far from the main village, and the health centre. We are less in contact with them and they also don’t come to the centre.*

Community health worker (Female, 28 years).

## Discussion

The findings from bi-variate analysis and adjusted logistic regression also reinforced from qualitative analysis indicate that preference for early child bearing is significantly associated with tribals in comparison to non-tribal. The current findings indicate that young married people from tribal community have higher odds of planning a child early within one year of marriage. These findings have also been recognised in earlier studies that have found more cases of early marriage and child bearing among tribal populations than other socio-ethnic groups [1, 2, 12]. Studies on tribal population in south and west India have also found low contraceptive use, misconceptions and lack of access to various contraceptive methods to be the causes of early child bearing [2, 14, 19]. The tribal populations have less education, which further makes them vulnerable to low contraceptive use and high unmet need than other social groups [12, 19]. The bi-variate association between tribal status and gender inequitable attitudes was also significant. Overall, tribals had greater gender inequitable and patriarchal attitudes on early childbearing as compared with their non-tribal counterparts.

This study contributes to a growing body of studies on reproductive behaviour and is one of the few studies using the GEM scale to understand gender-equitable attitudes of tribals and non-tribals. Existing studies have reflected gender equitable attitude for equitable reproductive decision-making and increased contraceptive use [20, 21]. However, such linkages are explored less among tribal populations. While historically gender relations among tribals have been more egalitarian as compared to other social groups, anti-female patterns of discrimination are now increasing among some tribal communities as their lives become integrated into mainstream culture and social practices, generally through the conversion to Hinduism [11, 15]. These studies have pointed out the growing trend of gender deficit of females and gender bias among tribal communities [11, 15].

Further, findings also indicate that tribals who were more likely to plan child early was attributable to inequitable gender norms associated with early child bearing. This reflects that not only do gender attitudes influence the perception of young married people among tribals for early child bearing but they also impact the planning of a child within one year of marriage. The present findings have reaffrmed this connection on a firmer footing. This is particularly noteworthy in light of the current findings. Gender attitudes of young married people were a cause of concern especially among tribal groups and therefore support the need for involvement of both men and women for addressing traditional gender inequitable norms for reproductive behavior. This finding also corroborates with a recent study conducted among tribal populations in rural India on reproductive behavior, where tribals were less likely to practice spacing contraceptive use (SCU) than their non-tribal counterparts. The lower SCU among tribal was driven by gender inequalities and social vulnerabilities (higher son preference, higher fertility preference and low education) [19].

The finding also confirms that the pressures for early child bearing among tribals fall more on young married women than men. Young married women had higher odds of planning a child within one year in comparison to their male counterparts. Qualitative findings congruently found that young married women were more influenced by negative prejudices around delayed childbearing and obliged to have at least one child before seeking family planning services. This was also found in previous studies in India [22]. The role of higher education in influencing fertility intention among non-tribal and tribal cannot be neglected, although it was positively associated with tribal status however not significant. The results reflect that higher education (above class 10th) matters in determining fertility behavior among young married people in both non-tribal and tribal groups. Qualitative accounts further demonstrated that young married people from tribal communities had less access to health services and modern contraceptives. There was also poor outreach of reproductive services by community health workers to tribal communities. A review also highlights that tribal people face geographical isolation and limited interactions and exposure, along with poor access to healthcare in India [8].

### Limitations of the study

The current findings should be viewed in context of certain limitations, which are important to state. The sample taken for the analysis was small (*n* = 273) and from the larger data on young men and women thus, the findings currently described may not hold true across other geographic and socioeconomic contexts and hence there can be limited generalisations. Further, the use of selective cross-sectional data limits our ability to make causal inferences at macro level. Therefore, further research will be required to extend and validate this analysis by including larger representative sample across different contexts.

## Conclusion

This paper reaffirms that efforts need to be directed towards the tribal populations the most. These findings call for pursuing gender transformative strategies for promoting gender equitable attitudes for reproductive decision making especially among tribals. The focus of health and population interventions should not only be to target young married tribals, but also to improve their gender attitudes for the purpose of delaying early child-birth. Efforts should also be intensified for providing access to higher education among tribals and non-tribals as it can significantly influence fertility intention. There is also a need to reduce disparities in access to family planning health services that exist between tribals and other social groups, strengthening outreach for family planning programmes. The findings are relevant to design and tailor appropriate family planning interventions for young married people to meet their current and future fertility desire needs.

## Abbreviations

AWC: Anganwadi Center
BPL: Below Poverty Line
FMR: Female-male ratio
GEMS: Gender Equitable Men’s Scale
IDIs: In-depth interviews
KIIs: Key informant interviews
NHFS: National Family and Health Survey
OBC: Other Backward Classes
OR: Odds Ratio
SC: Scheduled Caste
SCU: Spacing Contraceptive Use
ST: Scheduled Tribes

## Footnotes

1 Adolescence is defined by WHO as between 10 and 19 years, while youth refers to 15–24 years. Young people refer to the 10–24-year-old age group. For the purpose of this paper, we have used the term young people throughout to refer to 14–21 years of boys and girls in the study population.
